# Deep Learning-Based Phenotyping System With Glocal Description of Plant Anomalies and Symptoms

**DOI:** 10.3389/fpls.2019.01321

**Published:** 2019-11-14

**Authors:** Alvaro Fuentes, Sook Yoon, Dong Sun Park

**Affiliations:** ^1^Department of Electronics Engineering, Chonbuk National University, Jeonju, South Korea; ^2^Department of Computer Engineering, Mokpo National University, Muan, South Korea; ^3^College of Artificial Intelligence, Tianjin University of Science and Technology, Tianjin, China; ^4^Division of Electronics and Information Engineering, Chonbuk National University, Jeonju, South Korea

**Keywords:** deep learning, plant anomalies, recognition, glocal description, user-friendly information

## Abstract

Recent advances in Deep Neural Networks have allowed the development of efficient and automated diagnosis systems for plant anomalies recognition. Although existing methods have shown promising results, they present several limitations to provide an appropriate characterization of the problem, especially in real-field scenarios. To address this limitation, we propose an approach that besides being able to efficiently detect and localize plant anomalies, allows to generate more detailed information about their symptoms and interactions with the scene, by combining visual object recognition and language generation. It uses an image as input and generates a diagnosis result that shows the location of anomalies and sentences describing the symptoms as output. Our framework is divided into two main parts: First, a detector obtains a set of region features that contain the anomalies using a Region-based Deep Neural Network. Second, a language generator takes the features of the detector as input and generates descriptive sentences with details of the symptoms using Long-Short Term Memory (LSTM). Our loss metric allows the system to be trained end-to-end from the object detector to the language generator. Finally, the system outputs a set of bounding boxes along with the sentences that describe their symptoms using glocal criteria into two different ways: a set of specific descriptions of the anomalies detected in the plant and an abstract description that provides general information about the scene. We demonstrate the efficiency of our approach in the challenging tomato diseases and pests recognition task. We further show that our approach achieves a mean Average Precision (mAP) of 92.5% in our newly created Tomato Plant Anomalies Description Dataset. Our objective evaluation allows users to understand the relationships between pathologies and their evolution throughout their stage of infection, location in the plant, symptoms, *etc*. Our work introduces a cost-efficient tool that provides farmers with a technology that facilitates proper handling of crops.

## Introduction

Plant diseases are responsible for major economic losses in the agricultural sector worldwide (Martinelli et al., 2015). They are directly related to food safety and sustainable food production (Savary et al., 2012). Quantifying the impact of plant pathologies in crops represents one of the most challenging problems in agriculture (Donatelli et al., 2017). Nutrition deficiency or imbalance between soil moisture and oxygen makes a plant more susceptible to get affected by pathogens. Anomalies in the plants can be caused by pest, diseases or other abiotic stresses such as low temperature. The diseases recognition task is often related to time-consuming, laborious, and subjective. Traditionally, crop inspection has been carried out visually by people with some expert knowledge in the field. However, regarding any activity carried out by humans, this approach is subject to generate a degree of uncertainty or error (Barbedo, 2018a), and consequently, leads to incorrect decisions to control them. In addition, it is not always possible to control plant diseases, especially in remote areas with difficult conditions. On the other hand, it is also important to notice that the economic factor is also an inconvenience.

With the rise of media and technology, the application of deep learning-based methods has been increased widely. Along with that, fast and accurate approaches are rising in demand for better results. The application of this technology has been also extended to the area of plant diseases recognition. Several automated diagnosis methods (Kawasaki et al., 2015; Fuentes et al., 2016; Mohanty et al., 2016; Sladojevic et al., 2016; Amara et al., 2017; Fuentes et al., 2017a; Fuentes et al., 2017b; Araus et al., 2018; Ferentinos, 2018; Fuentes et al., 2018; Liu et al., 2018; Barbedo, 2018b) have been proposed to detect plant diseases and pests in different types of crops. They offer an effective tool for people who are involved in the agricultural area. An accessible application of this technology can offer even more possibilities to farmers in different parts of the world who lack the advanced technology to manage their crops in an appropriate way so that they can avoid such economic losses (Ferentinos, 2018). This technology can be adapted for large-scale cultivation, and represents a tool to monitor breeding programs efficiently in real-time (Fujita et al., 2016).

**Figure 1:**
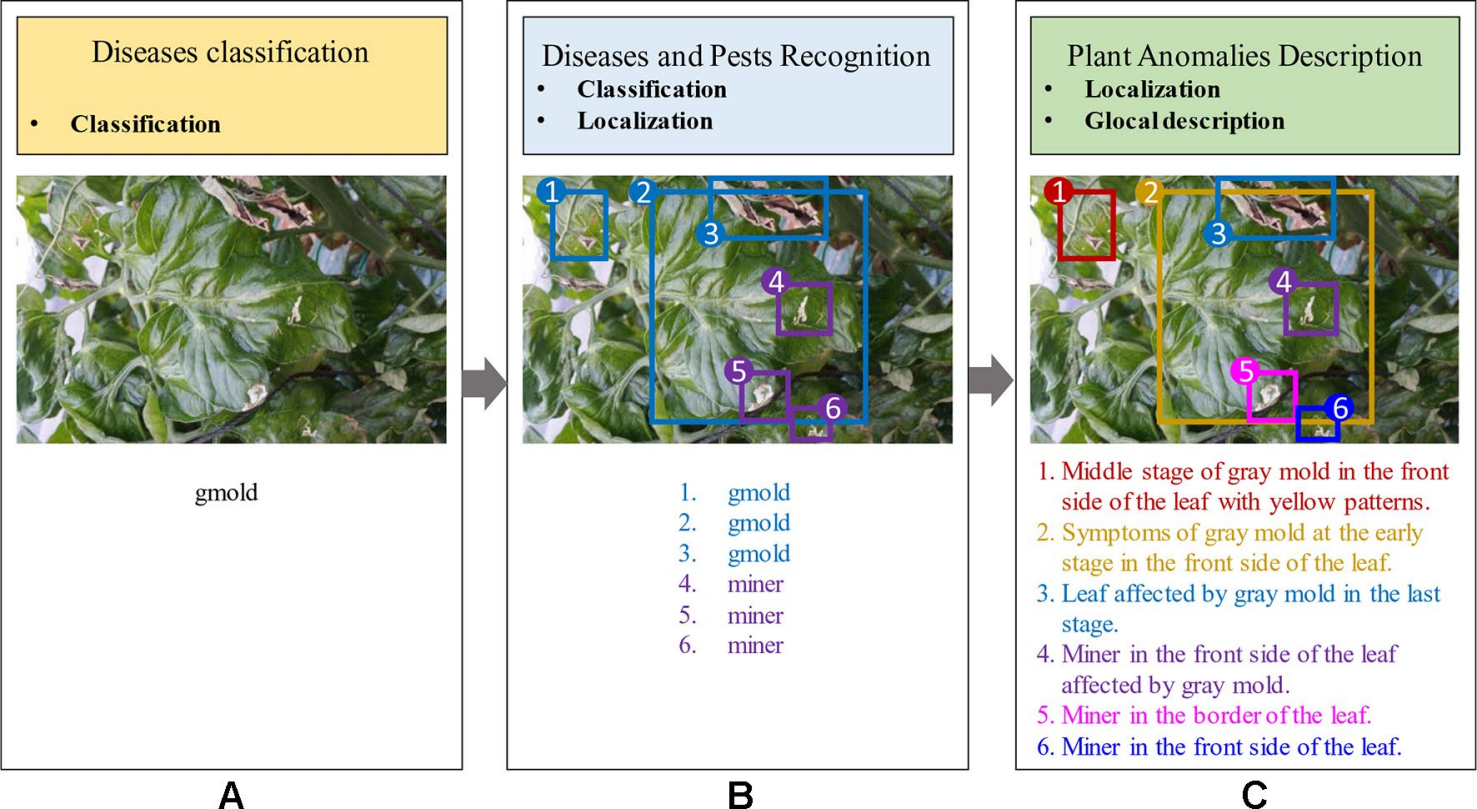
General idea of frameworks for Plant Diseases Recognition. **(A)** Image-based diseases classification (class). **(B)** Region-based diseases and pest recognition (class, localization). **(C)** Our proposed approach for plant anomalies description (localization, glocal description). The proposed model provides more detailed information about anomalies that affect the plants and their interactions in the scene.

Although recent studies in plant anomalies recognition have shown some progress, the accuracy of these frameworks greatly depends on the extraction and selection of the visible characteristics of the disease. Specifically, regions containing the infected area of the plant should be extracted. To address that problem, two different ways have been commonly used: 1) Image-classification-based diseases detection (Mohanty et al., 2016), and 2) Region-based diseases and pest recognition (Fuentes et al., 2017b; Fuentes et al., 2018). The first approach estimates if an image contains any instances of an object class (what), while the second one, provides information about the class and location instances of any particular object in the image (what and where). Both methods present limited capabilities in providing a real estimation of the symptoms of diseases in plants. The proposed system aims to provide more user-friendly information that allows people to better understand the state of the crop. Consequently, we introduce a method that combines the capabilities of the previous approaches, and on the other hand is able to generate more detailed information about the plant anomalies and their symptoms. **Figure 1** shows a visual representation of the aforementioned approaches.

In this work, we take a step towards deep learning tools and propose a system that generates a user-friendly estimation of plant anomalies, specifically tomato plants (Cruz et al., 2017). We further investigate the interaction between anomalies along with their inter- and intra-class variations. More specifically, the goal of our approach is to automatically localize and describe anomalies in tomato plants using a “glocal” concept, which generally represents the interconnection between global and local regions. Conceptually, in our approach, global represents regions of the context and local introduces the specific areas of the anomalies in the image.

For the purpose of our research, we identify the following main challenges: First, a detector should be able to provide the class and location of the anomalies in the plant. Second, a language generator should associate each region of the image with its corresponding text. Third, the information from both should be then combined to generate descriptions of the anomalies in the plant in the form of fully comprehensible sentences. Each sentence is further associated with a specific region (bounding box) of the image and also provides a reliable description that includes surrounding objects, infection status, patterns, colors, *etc*. Using the glocal criteria, a description is a statement of the symptoms and their characteristics of the plant. **Figure 1C** illustrates the purpose of our work.

To address the above points, in this paper, we introduce a technique that explores the learning capabilities of Deep Neural Networks (DNN) for object detection and Recurrent Neural Networks (RNNs) for text generation, and propose an automated diagnostic system for plant anomalies recognition. Our system consists of two parts: First, a detector is trained to obtain a set of region features that contain plant anomalies using a Region-based Deep Neural Network. Second, a language generator takes the features of the detector as input and generates descriptive sentences with details of the symptoms using Long-Short Term Memory (LSTM). In addition, we generate a single sentence that describes the scene in general. Our loss metric allows the system to be trained end-to-end from the object detector to the language generator. Finally, the system generates a set of bounding boxes along with specific sentences that describe the symptoms.

The main contributions of our work are summarized below: 1) We propose an end-to-end system that besides being able to detect plant anomalies and their location in the image, allows to generate more detailed information about their symptoms and interactions with the scene. It uses an image as input and generates a diagnostic result as output. We further obtain specific descriptions of the contents in the image (local) and an abstract description of the scene (global). 2) Our loss metric allows the system to be trained end-to-end from the object detector to the language generator. 3) To improve stability and results, we further implemented some additional knowledge added to the language generator, such as context information and fusion techniques. 4) We demonstrate the efficiency of our approach using our newly created tomato plant anomalies description dataset. We collected the images of our dataset in real conditions of plant environments, using cameras with different resolutions. In addition, we designed an annotation procedure based on the glocal concept. We annotate plant anomalies in the images using bounding boxes along with their specific descriptions using sentences. The insights drawn from the experimental results led to a better understanding of the strengths and limitations of plant anomalies recognition. Our results suggest some potential targets for future research on the subject as it constitutes an efficient tool to monitor the state of plants.

The remainder of the paper is introduced as follows. In *Related Works Section*, we review techniques used for object detection and recognition, language generation and plant diseases recognition. Our proposed system is presented in *Phenotyping System With Global Description of Plant Anomalities and Symptoms Section*. We then evaluate the performance of the system through the experimental results in Experiment Results (Section). Finally, we conclude this work in *Conclusion Section*.

## Related Works

In this section, we first introduce some related works based on deep neural networks for object detection and image description. Then, we review some recent techniques used for plant anomalies recognition.

### Deep Learning Methods for Object Detection and Image-Based Description

In vision systems, object detection has opened a wide range of opportunities with several applications in different fields. These systems involve not only recognizing and classifying objects in the image (Russakovsky et al., 2015) but also localizing them by drawing bounding boxes around their area (Ren et al., 2016). State-of-the-art methods based on deep learning for object detection can be categorized into two types: two-stages (Dai et al., 2016; Ren et al., 2016; He et al., 2017) and single-stage (Redmon et al., 2015; Liu et al., 2016; Redmon and Farhadi, 2017). Correspondingly, in recent years, much of the progress in deep learning has been also directed to develop handful and efficient feature extractors (Krizhevsky et al., 2012; Simonyan and Zissermann, 2014; Szegedy et al., 2015; He et al., 2016; Huang et al., 2017; Hu et al., 2017; Xie et al., 2017). Lately, Feature Pyramid Network (FPN) (Lin et al., 2017) has shown progress, especially in the recognition of objects at various scales. Basically, it exploits a pyramidal form of CNN feature hierarchy while creating a feature pyramid that has semantics at all scales. The result is a feature pyramid that has a rich semantics at all levels and is built quickly from a single input image.

In addition to recognizing patterns within images, methods based on deep learning have shown remarkable abilities to generate text as well (Bahdanau et al., 2015). In practice, to generate an automatic description from the images, it is necessary to understand how humans describe an image (Bernardi et al., 2016). Humans by nature have the ability to find relationships between objects and their possible interaction, their attributes and actions they perform. The problem of generating descriptions from visual data has been widely studied and recent interest has been put into solving the problem of image description in natural language (Kiros et al., 2014a; Kiros et al., 2014b; Donahue et al., 2017; Kaparthy and Li, 2017; Vinyals et al., 2017). For instance, Kiros et al. (2014a) used a CNN to learn representations of words and image characteristics together by jointly training a language model. Subsequently, Kiros et al. (2014b) proposed an encoder-decoder based method that learns a joint image-sentence embedding where sentences are encoded using LSTM recurrent neural networks. To that purpose, image features extracted by a CNN are projected into the space of the LSTM to generate language. Kaparthy et al. (Kaparthy and Li, 2017) developed a deep neural network that infers the alignment between segments of sentences and the area of the image that they describe. Specifically, they use a Region-Based Convolutional Neural Network (R-CNN) to find objects in the image, and a RNN to generate a description in the form of text. In addition, to make the combination of visual recognition and description end-to-end trainable, Donahue et al. (2017) proposed Long-term Recurrent Convolutional Networks (LRCNs). Further, Vinyals et al. (2017) introduced an end-to-end approach to generate a description of images. They combine vision (CNN) for image classification and language models (RNN) for language generation.

Although the detection and recognition of objects are necessary, they are not sufficient to produce detailed information. The results are a list of labels corresponding to the objects in the image. in specific applications, an efficient image description should not only contain a list of objects but also possibly a clear and concise description of them. In that direction, several recent studies take advantage of image description on regions to describe images with natural language (Johnson et al., 2016; Kaparthy and Li, 2017; Yang et al., 2017). They are specifically based on a combination of RNN language model that is conditioned on the image information. However, in those approaches, they tackle the problem from a subjective point of view, since they find the objects that are presented in the image but not a relationship between them. In our work, we extend the idea of object-based description as an application for recognizing plant anomalies. The system can provide more precise and clear information about the pathology that affects a plant.

### Plant Anomalies Recognition

The worldwide accessibility to mobile systems and the recent advances in software and hardware technologies have allowed the implementation of more efficient technologies in several areas. Recently, several works have demonstrated the potential and possibilities of utilizing deep neural network techniques for phenotyping in plants (Mutka and Bart, 2015; Singh et al., 2016; Araus et al., 2018; Singh et al., 2018). The rapid growth of sophistication and capabilities of deep neural networks have opened up a wide range of opportunities to extend their application towards the solution of common problems in the plant science research community, such as the case of plant diseases recognition. Following this trend, recent studies based on deep learning, have addressed automated identification of plant diseases by non-destructive methods in different types of crops. These methods can be divided into two types: image-based diseases recognition and region-based diseases recognition.

In approaches based on image classification, features of images containing a specific disease are extracted using CNN and subsequently classified into different categories. Some examples include the detection of plant anomalies in several crops such as apple (Liu et al., 2018), bananas (Amara et al., 2017), cucumber (Kawasaki et al., 2015), tomato (Fuentes et al., 2017a), *etc*. This application has been further extended to multiple crops (Mohanty et al., 2016; Sladojevic et al., 2016) to distinguish different types of pathologies out of healthy leaves. However, it is worth to mention that, although these approaches show the use of CNN-based methods as a powerful tool to extract features and efficiently classify images that contain particular diseases in different types of crops, they are limited to perform experiments using images obtained in the laboratory, rather than a real scenario. Therefore, they do not cover all variations included in real-field conditions such as state of infections, presence of various anomalies in the same sample, surrounding objects, *etc*. Consequently, their results may be subjective to the scene instead of the diseases in particular.

In contrast to the aforementioned works, Fuentes et al. (2017b) proposed a robust system that can recognize nine different types of anomalies in tomato plants. They show a satisfactory method that is able to provide the category (class) and location (bounding box) of pathologies using images collected in real-field scenarios. Recently, Fuentes et al. (2018) extended their work in (Fuentes et al., 2017b) and showed a significant improvement in the task of tomato plant anomalies recognition using a secondary diagnostic function based on CNN-filter banks to reduce the influence of the false positives generated by the detector. Compared to their previous approach (Fuentes et al., 2017b), they obtained a recognition rate of approximately 96% which is a gain of 13%. This system has also demonstrated to be an effective technique to address the problem of class imbalance that appears especially in datasets with limited data.

In general, although the works mentioned above have substantially allowed satisfactory detection and recognition of plant anomalies, they present limited capabilities to provide a better characterization of the problem, especially in real-field scenarios. In other words, they lack specific information that can specifically allow users to better understand the state of the infection based on the symptoms of diseases. To address this limitation, we propose an approach that differs mainly from previous methods in that, besides being able to detect plant anomalies and their location in the image, it also provides more detailed information about their symptoms and interactions with the scene. It uses an image as input and produces a user-friendly diagnostic result that is shown in the form of sentences as output.

## Phenotyping System With Glocal Description of Plant Anomalies and Symptoms

### System Details

**Figure 2:**
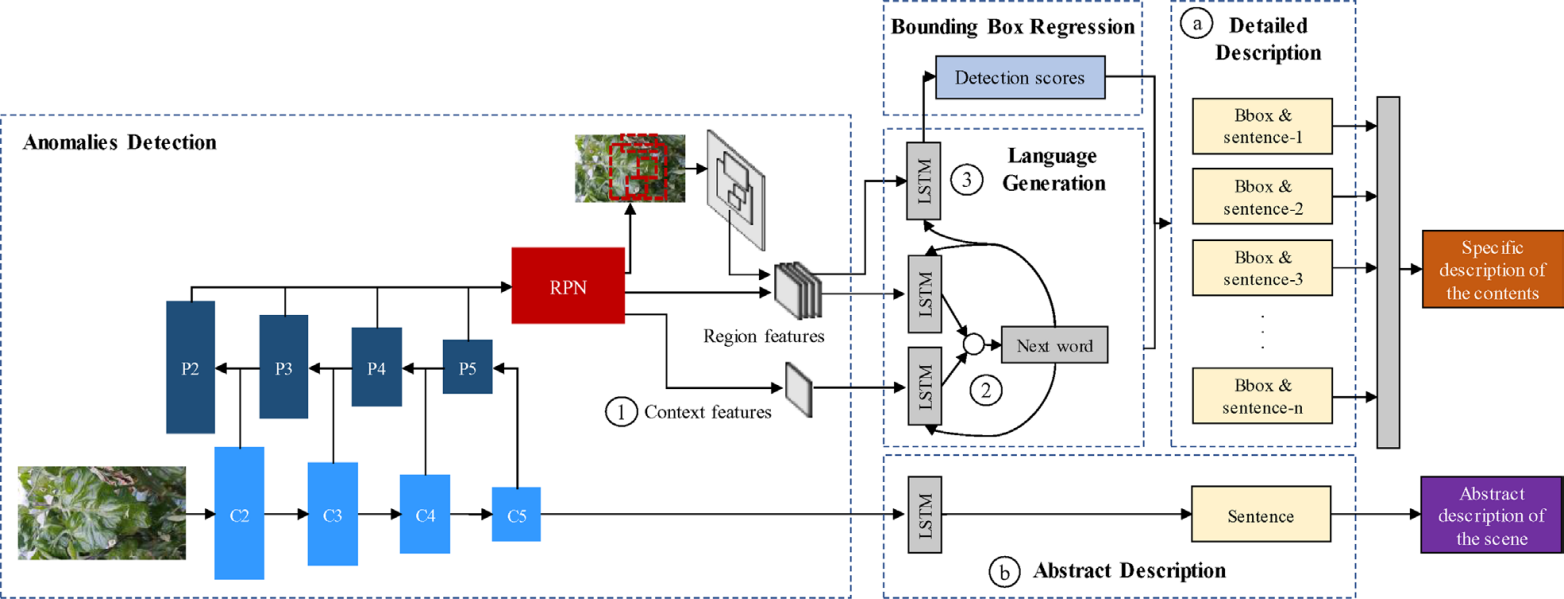
System overview of the proposed approach. It includes the object detection, language generation, localization, detailed description of the contents, and the abstract description of the scene. ⓐ Bounding boxes and detailed description of anomalies. ⓑ Abstract description of the scene. ① Context features obtained from the feature extractor. ② Region-context fusion. ③ Language-localization fusion.

The goal of our work is to propose a system that generates glocal descriptions of anomalies in the plant. Our system consists of two parts: First, an object detector is trained to obtain a set of region features that contain plant anomalies using a Region-based Deep Neural Network. Second, a language generator takes the features of the object detection results as input and generates descriptive sentences with details of the symptoms using Long-Short Term Memory (LSTM). Finally, the system outputs a set of regions of interest (bounding boxes) and their corresponding descriptions of the symptoms (sentences). Additionally, we generate a single sentence describing the scene in general. An overview of the proposed system is represented in **Figure 2**. We describe the contents of our work in the following subsections.

#### Plant Anomalies Detection *vs* Anomalies Description

**Figure 1** shows a general overview of the idea pursued by our proposed framework. To demonstrate the capabilities of our system, we firstly differentiate between image-based anomalies classification (**Figure 1A**), region-based anomalies recognition (**Figure 1B**), and plant anomalies description (**Figure 1C**). In general, the first approach provides a holistic image classification (what) which sometimes is limited to the salient objects of the image. Given one target class that corresponds to the whole image can be quite subjective to the representation. In the second approach, an image can provide a better interpretation of the contents. It provides information about the class and location instances of any particular object in the image (what and where). On the other hand, the third approach, which is in the scope of our work, is able to not only localize plant anomalies in the image but also provides more detailed information about the symptoms using sentences. In addition, this local description is able to objectively represent the visual elements and their relationships between them.

### Recognition and Description of Plant Anomalies

The main idea of our approach is to incorporate visual and word features to locate and describe the symptoms of anomalies that affect plants. Our system, in general, introduces a tool that provides more specific information about the state of the plant. The input to our system is an image *I* and the output is a set of sentences *Si* that especially describe the symptoms happening within a specific area of the plant. Each description consists of several words *y* that fit a fully understandable sentence.

(1) $$S_{i}=\left\{y_{1},y_{2},\ldots ,y_{C}\right\},\;\;y\in {\mathbb{R}}^{k},\;\;\;i=1,2,3,\;\ldots ,n\;$$

where *k*, *c* and *n* represent the size of the vocabulary, length of the sentence and number of bounding boxes in the image respectively.

Considering the general idea of the system, our baseline model begins with two main parts: an object detector and a language generator. Each part performs a function that allows the system to describe the contents of the image using an end-to-end training manner. However, we expand its capabilities so that the system can perform “specific descriptions of the anomalies” and an “abstract description of the scene”. We explain each part below.

#### Specific Description

The purpose of this part is to generate detailed information about the plant anomalies, specifically including their symptoms, patterns, color, location, infection status, *etc*. Each detected region is associated independently with a specific sentence describing its behavior (See ⓐ in **Figure 2**).

##### Anomalies Detection

This part is responsible for detecting regions that contain anomalies in the plant. For this, we extend the application of Faster R-CNN (Ren et al., 2016). It uses a two-stage process to detect objects in the image. In the first stage, a Region Proposal Network (RPN) takes the feature maps of an image as input and outputs a set of object proposals with their region score. In the second stage, the feature vectors of the object proposals are fed into a network to predict the localization of the bounding boxes.

Our application, in particular, contains several visual variations such as the stage of infection, color, patterns, location, occlusion, and especially objects at various scales, *etc*. Therefore, the object detection part should be efficient enough to achieve satisfactory detection results while dealing with all variations in the image. A special difficulty of object detectors is the capability to detect small-scale objects. Therefore, we propose to use a pyramid-based architecture as the feature extractor of the anomalies detector. This is represented in the feature extractor of **Figure 2**.

FPN (Lin et al., 2017) is one of the most recent CNN architectures that have shown progress, especially in the recognition of objects at various scales. Basically, it exploits a pyramidal shape of a CNN feature hierarchy while creating a feature pyramid that has semantics at all scales. The result is a feature pyramid that has rich semantics at all levels and is built quickly from a single input image. It produces proportionally sized feature maps at multiple levels. This process is independent of the feature extractor used as the backbone. In our paper, we use a ResNet-50 network as the basis of the FPN. The ResNet-50 network consists of five residual blocks. The FPN takes the output of the last layer of each block to construct the pyramid using a bottom-up pathway. The outputs of the last residual blocks are denoted as {*C_2_*,*C_3_*,*C_4_*,*C_5_*} or conv2, conv3, conv4, and conv5 outputs. Then, starting at the coarsest level, involving a top-down pathway, the FPN obtains higher-resolution features by spatially up-sampling the coarse levels to semantically stronger higher levels of the pyramid. These features are merged *via* lateral connections between levels. The sampled map is merged with the map of the next level, iteratively until the finest level of the pyramid. The final set of feature maps of the FPN are denoted as {*P_2_,P_3_,P_4_,P_5_*} as represented in **Figure 2**.

To be able to use a FPN network with Faster R-CNN, the system needs to compute Regions of Interest (RoIs) of different scales in the pyramid levels. Following the concept of an image pyramid, a FPN needs to assign an RoI of width *w* and height *h* to the level *P_k_* of the pyramid by:

(2) $$k=\left[k_{0}+\log_{2}\left(\sqrt{\mathrm{wh}}/224\right)\right]$$

where, *k_0_* is the target level on which the RoI should be assigned, and 224 is the pre-training model size of the image. Our FPN is composed of four levels which are extracted from the residual blocks of the ResNet. Consequently, the scale of the RoIs becomes smaller at finer-resolution pyramid levels and prediction is performed at all levels of the pyramid. Based on these statements, we are able to train the detector to obtain regions that contain plant anomalies.

##### Bounding Boxes Localization

Having computed the feature vectors of the regions containing anomalies of the plant using the first stage of Faster R-CNN, the localization part predicts the coordinates of the bounding boxes using Non-Maximum Suppression (NMS) and Region of Interest (ROI) pooling layer. See the region features in **Figure 2**.

##### Language Generation

The same region features generated by the object detector, are used as inputs of the language generator that associates each region with text. In this part, LSTM modules predict words at each time step and use those predictions to predict the next word from the init token until the end of the sentence. This procedure is shown in the language generation part of **Figure 2**. LSTMs are a special kind of units of RNNs that incorporates a built-in memory cell to store information and exploit long-range context (Hochreiter and Schmidhuber, 1997). They are able to learn long dependencies while avoiding the problem of long-term dependency. In addition, when integrated with image models, LSTM systems are not limited to fixed-length inputs or outputs, allowing simple modeling for sequential data of different lengths, such as machine translation, speech recognition. However, different from text or video, our approach overcomes the problem of region description using a single input region and expecting a label space consisting of sentences with various lengths.

Our language generator model takes the region features of the image *I* hat are generated by the RPN of the object detector and the sequence of input words {*x*_1_,*x*_2_,...*x_C_*} The LSTM then computes a sequence of hidden states {*h*_1_,*h*_2_,...*h_C_*} or each word and a sequence of outputs {*y*_1_,*y*_2_,...*y_C_*} y the following relation for all words:

(3) $$b_{v}=W_{\mathrm{hi}}\left[R_{\theta c}\left(I\right)\right]$$

(4) $$h_{c}=f\left(W_{\mathrm{hx}}x_{c}+W_{\mathrm{hh}}h_{c-1}+b_{h}+1⊙b_{v}\right)$$

(5) $$y_{c}=\mathrm{classifier}\;\left(W_{\mathrm{oh}}h_{c}+b_{o}\right)$$

where, *W_hi_*, *W_hx_*, *W_hh_*, *W_oh_*, *b_h_*, *b_o_* are the learnable parameters, *R_θc_* (*I*) epresents the set of region features, and *b_v_* *i*s an image context vector extracted from the last feature map of the CNN. The output vector *y_c_* generates the probable words included in the vocabulary and an additional dimension to finish the LSTM model. The RNN model has a final size of 512 neurons.

Starting with the first word of the sentence *y*_1_ and the desired known word *x*_1_ the network predicts the new word *y*_2_ until the last word *y_c_* in the sentence. The final goal of the LSTM is to find words with the higher probability using a classifier, such as *softmax*.

#### Abstract Description

An abstract description provides general information about the symptoms and their interactions with the scene. Its main purpose is to provide a broader view of the anomalies that affect plants considering both global and local information. The first is obtained from the features of the input image while the second is generated by the object detector and the language generator (See ⓑ in **Figure 2**).

To obtain the global features of the scene, we use the same ResNet-50 architecture as in the object detector. Next, the abstract description of the scene is obtained by applying of an independent LSTM module that uses the last feature map of the CNN as input. A representation of the abstract description part is shown in **Figure 2**.

### Accurate Localization and Description

As shown in **Figure 2**, our model can generate descriptions of the regions included in the input image. However, in order to improve the performance, we further analyze some of the specific components included in the architecture. Our main criteria about the capabilities of the system lie into the following facts:

- Context features: The use of context features provides information about the main scenario of the image (See ① in **Figure 2**).
- Region-context fusion: The use of LSTM modules and fusion techniques for the region and context features helps to improve the accuracy of the language generator (See ② in **Figure 2**).
- Language-localization fusion: The use of an LSTM module to match the vocabulary and the region features can improve the localization of the bounding boxes (See ③ in **Figure 2**).

To understand the capabilities of these criteria, we mainly focus in the language generation and localization. The first component is found in the use of “context information” in the language generator. This recalls a second component, which is the way to “fuse” the context information with the regions generated by the RPN. These are, a) early fusion, using a single LSTM module or, b) late fusion, using independent LSTM modules. Finally, a third component associates each bounding box with specific words in the sentences that have been generated using the region and context features.

### Model Complexity

The complexity of the system is represented in terms of the number of parameters of both the feature extractor used by the object detector, as well as the LSTM model for the language generator. These are 24,576,000 for the feature extractor, and 125,050,075 for the LSTMmodel. We use a ResNet-50-based FPN network (Lin et al., 2017) as the baseline architecture of our approach.

### Training

The model is trained end-to-end and consists of three main steps: 1) Detection of anomalies; 2) Detailed description; 3) Abstract description. In the first part, a Region-based Neural Network extracts the region features from the image. Next, the LSTM modules generate specific language descriptions that associate words with regions of the image. Finally, the abstract description is generated using the context features of the input image and an independent LSTM module.

Training the complete model end-to-end aims to minimize the following loss function:

(6) $$L=L_{\mathrm{spec}}+L_{\mathrm{abs}}+\lambda L_{\text{det}}+\beta L_{\mathrm{reg}}$$

where, *L_spec_*, *L_abs_*, *L_det_*, and *L_reg_*, are the specific description loss, abstract description loss, detection loss, bounding box regression loss respectively. *λ* and β are weight hyperparameters. *L_spec_* and *L_abs_* corresponds to cross-entropy calculated for text prediction at each time step. *L_det_* and *L_reg_* re calculated similarly as in the Faster R-CNN (20). The detection loss is a cross-entropy two-class loss for foreground and background, while the bounding box regression loss is a Smoothed-*L*1 loss.

## Experimental Results

### Tomato Plant Anomalies Description Dataset

#### Images

To validate our experimental results, we used the images from our Tomato Diseases and Pest dataset (Fuentes et al., 2017b; Fuentes et al., 2018). Our dataset consists of approximately 5,000 images collected from farms located in different areas of South Korea. The images were taken under different conditions and scenarios. They include anomalies that can be developed depending on the season and variables such as temperature and humidity. Since not all diseases can be found throughout the year, but in seasons, the number of images corresponding to each class is different. The categories and the number of annotated samples used in our system can be seen in **Table 1**. The number of annotated samples represents the bounding boxes annotated in the images after data augmentation. Every image contains several samples based on the affected areas of the plant. The background class is collected as a transversal category that is annotated in most of the images.

**Table 1:** List of categories in our tomato diseases and pests dataset and number of annotated samples.

Class	Number of Images in the Dataset^1^	Number of Annotated Samples (Bounding Boxes)^2^	Percentage of Bounding Box Samples (%)
Leaf mold	1,350	11,922	24.06
Gray mold	335	2,768	5.57
Canker	309	2,648	5.33
Plague	296	2,570	5.17
Miner	339	5,283	10.63
Low temperature	55	477	0.96
Powdery mildew	40	338	0.68
Whitefly	49	404	0.81
Nutritional excess	50	426	0.85
Yellow leaf curl	3,927	3,927	7.90
Background^3^	2,177	18,899	38.03
Total	8,927	49,662	100

^1^Number of images in the dataset;

^2^Number of annotated samples after data augmentation;

^3^Category included in every image.

#### Groundtruth and Vocabulary

Since the purpose of our approach is to generate sentences that describe the symptoms of plant anomalies, we have taken the images of the Tomato Diseases and Pest dataset to create our new Tomato Plant Anomalies Description Dataset.

Differently from our previous dataset, our new dataset contains three types of information that have been annotated in every image: a) Coordinates of the bounding boxes showing the location of the anomalies, b) Detailed descriptions of the anomalies shown within the bounding boxes and, c) Abstract description of the scene. These facts provide user-friendly information while offering a more realistic representation of the scene and the anomalies that affect the plants.

**Figure 3:**
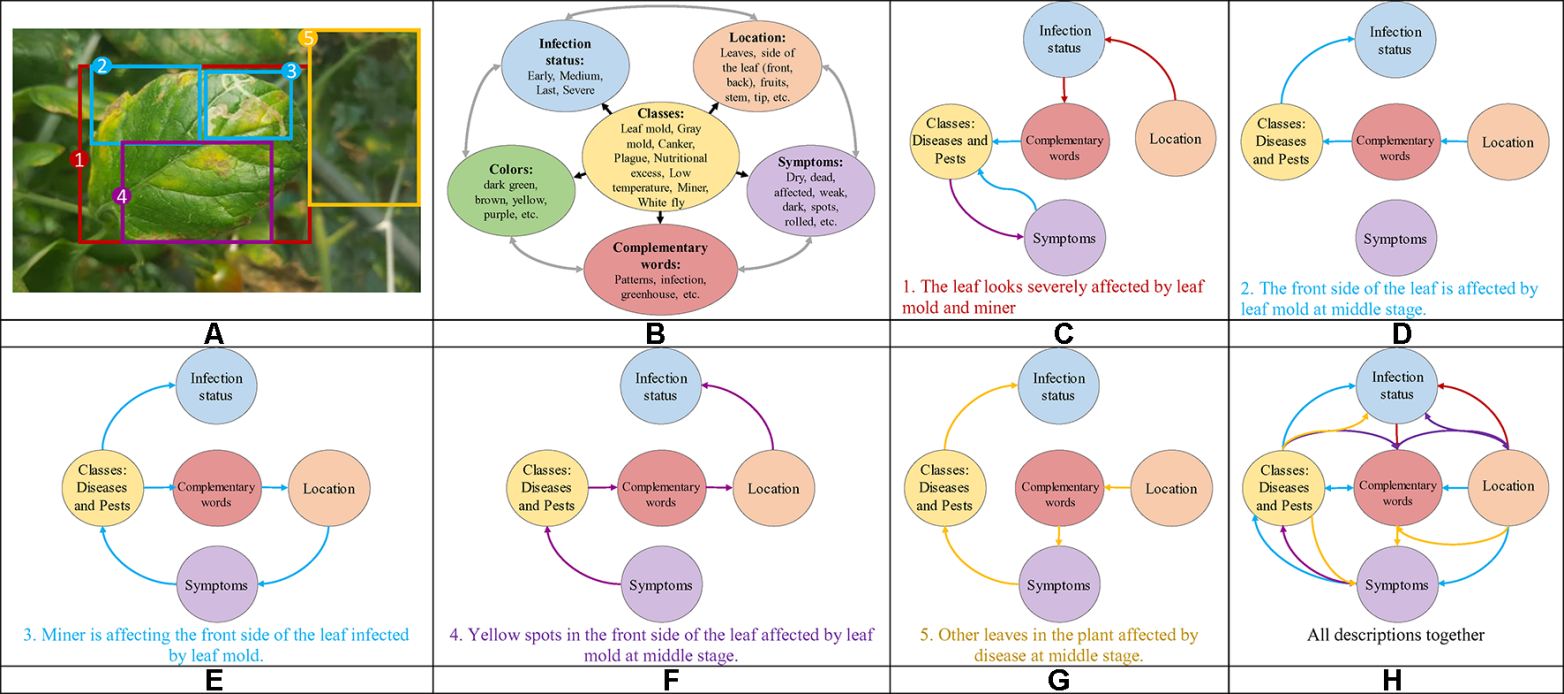
Example of the procedure used to annotate the images in our Tomato Plant Anomalies Description Dataset. Each sample in dataset includes: **(A)** Coordinates of the bounding boxes. **(B)** A representation of the specific terms included in our annotations and their relationships. **(C**–**H)** Specific descriptions of the symptoms and abstract description of the scene. Note that each color of the sentences represents the corresponding bounding box in the image and information such as Global, Local, Attributes, and Background. To describe the symptoms, each sentence combines information from the variables such as classes, symptoms, location, infection status, and complementary words.

Our research presents certain characteristics that make it different from a simple generation of sentences to describe an event. A key aspect that we have mainly taken into account is the design of the process used to annotate the images of our dataset. We have paid special attention not only to plant anomalies but also to the relationship between them and the scene. For instance, we considered cases when two or more anomalies are presented in the same sample image and located in different parts of the plant such as front or back sides of the leaves. **Figure 1C** shows some sample annotations. In this sense, we follow the procedure below to annotate each of the images in our database:

- We identified the anomalies and obtain their localization coordinates in the image using bounding boxes. We selected the anomalies using the glocal criteria, which represents the following regions: global, local, attributes, and background. We defined at least one global and more local samples. The attributes represent the patterns, colors, *etc*., and the background localizes other regions in the image. **Figure 3A** shows the types of information and their location in the image.
- We identified the key terms and their relationships to generate a sentence that accurately describes the anomalies. To determine these terms, we first considered the following characteristic: classes (types of anomalies), symptoms, location, infection status, and complementary words. We looked for terms that may have a relationship with our specific task. **Figure 3B** represents some terms used for our purpose, which are divided into each category.
- Once identified the regions that show specific anomalies in the plant, we described their symptoms with a sentence that contains key terms and connecting words. Starting with a specific term from our categories, each sentence is constructed as a combination of words that describe a symptom and its relationship with the scene through the interaction between global, local, attributes and background regions. For example, **Figures 3C–G** show the paths and combinations to generate descriptions of the regions. The lines in color represent the interactions between regions. Some cases include, for instance: local regions that show different symptoms (**Figures 3D–E**), attributes of the anomaly at a specific infection status (**Figure 3F**), as well as other regions in the image that can be also affected by anomalies (**Figure 3G**), etc. The global region describes the interactions of several locals along with their attributes (**Figure 3C**).

In addition, it is necessary to mention that this procedure allows users to better understand the interactions between anomalies and with the scenario, as shown in **Figure 3H**. Finally, the annotation of each image includes a set of bounding boxes, detailed and abstract descriptions of the anomalies.

**Figure 4:**
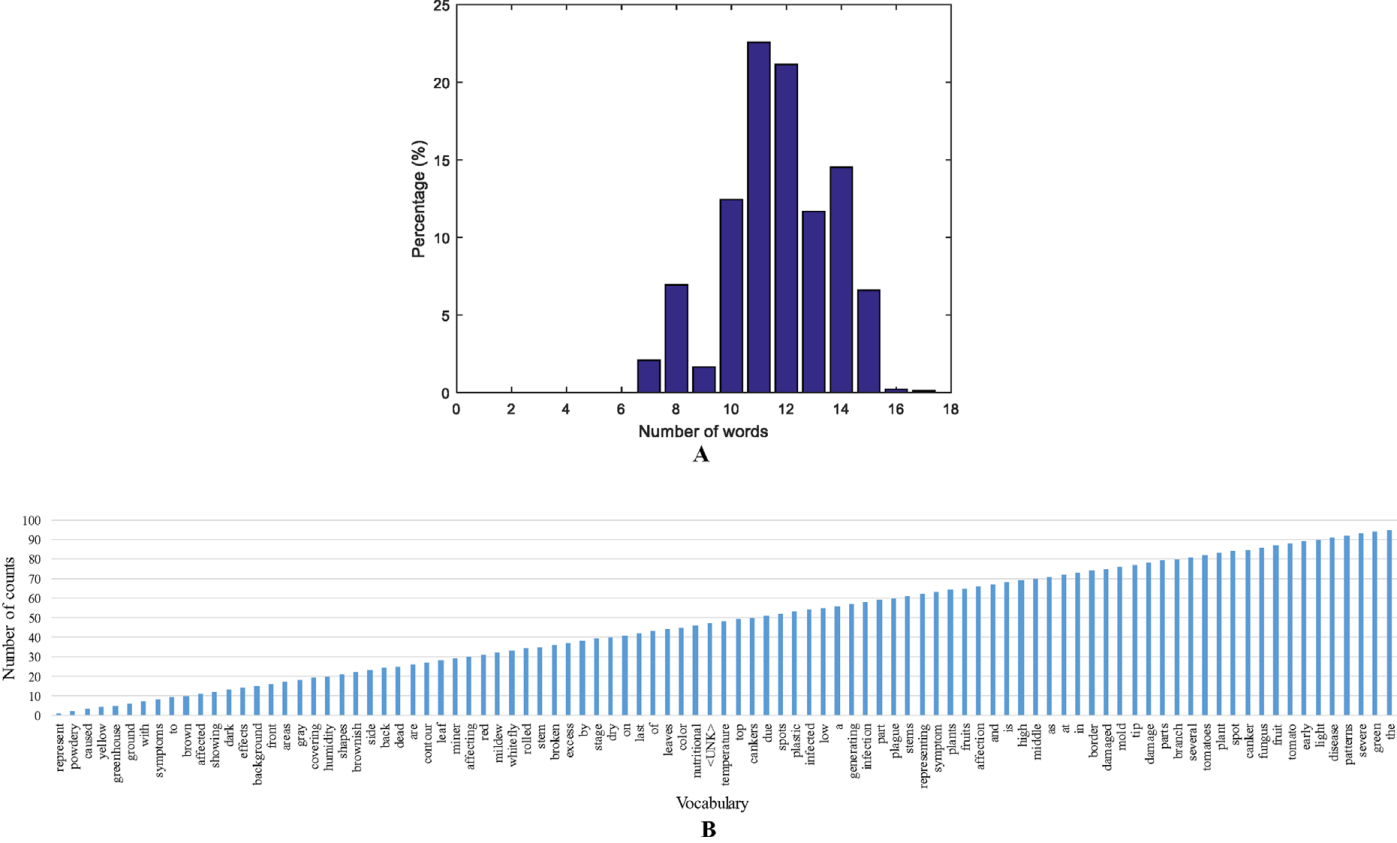
A representation of the vocabulary in the dataset. **(A)** Sentence length distribution based on the number of words. **(B)** A representation of the vocabulary used in our approach.

We annotated each image in the dataset and obtained a vocabulary of words. Every encoded-word in the sentences is extracted to build the vocabulary and obtain statistics such as top words, and sentence length. We further determined the statistics of the sentence length by counting the number of words that include the target class and connecting words. Our dataset is mostly composed of sentences with 11 and 12 words, as shown in **Figure 4A**.

Additionally, we found the most used words in the vocabulary by counting the number of repetitions. Our vocabulary is mainly composed of connective words, verbs, and nouns, as well as, the target anomalies or other type of stress. Some classes with more number of samples have more influence on the top words and repetitions in the vocabulary. A representation of the statistics of top words and the vocabulary used in our approach is presented in **Figure 4B**.

### System Training and Validation Details

The system has been trained end-to-end using a PC computer equipped with two NVIDIA Titan XP GPUs. The dataset has been divided into 80% training, 10% testing, and 10% validation. The complexity in terms of the number of parameters of the feature extractor and the language generator LSTM is mentioned in *Model Complexity Section*.

To improve the results and stability during training, we used transfer learning from a pre-trained model in the ImageNet dataset. The training weights are initialized with Adam optimizer. In addition, we used extensive data augmentation to increase the number of samples and avoid overfitting problems.

### Evaluation Metric

In general, our system uses a single image as input and generates a set of regions along with their descriptions. Thus, we designed a system that is able to effectively predict plant anomalies (anomalies detector) along with their localization coordinates in the image (bounding boxes) and accurate descriptions (language generator). To evaluate the performance of the system, we used the mean Average Precision (mAP) as our metric. The mAP measures both localization precision and language accuracy. In the localization, we evaluated the Intersection-over-Union (IoU) with a threshold of 30%, and to measure language similarity we used METEOR (Banerjee and Lavie, 2005) with a threshold of 25%. METEOR is an automatic machine translation evaluation which measures the correlation between the metric scores and human-produced reference translations. We evaluated the average precision using the two methods mentioned above to report the mAP in the following quantitative results.

### Quantitative Evaluation

**Figure 5:**
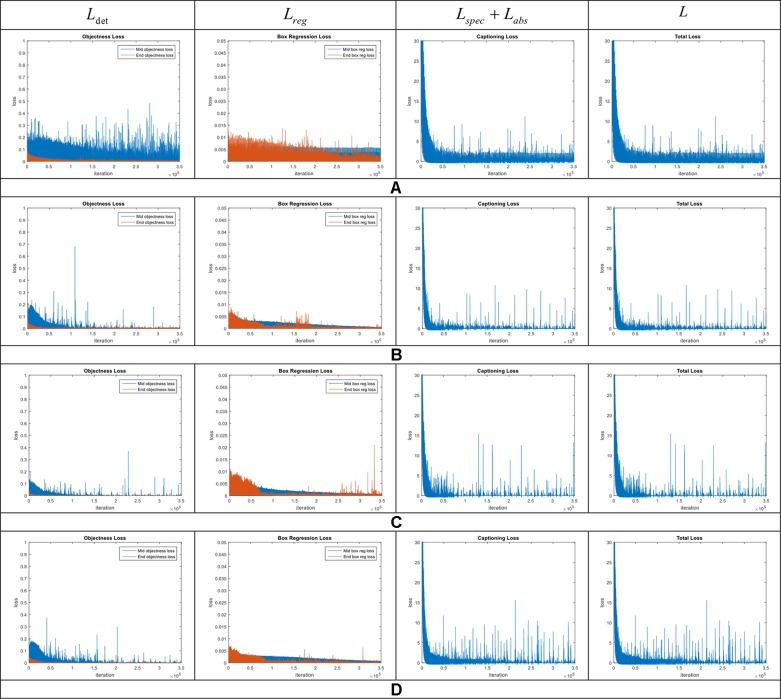
Loss curves in the model introduced in our approach. Each row represents the curves of the components included in the general framework. **(A)** Model without context features. **(B)** Region-context features early fusion. **(C)** Region-context features late fusion. **(D)** Complete model, which also includes the language-localization fusion. Note the changes especially in the bounding box regression loss. The legends are represented by the middle and end losses. Middle means the evaluation at the middle layers and End means at the end of the network.

We evaluated the performance of our model and the added components to improve localization and description. To further clarify the effectiveness of the proposed model, we conducted experiments on our dataset. We show these results in the training curves of **Figure 5**. These include the components of the loss function (Equation 6) such as captioning loss, object detection loss, bounding box regression loss, and the total loss. To this effect, we trained the model end-to-end including the object detector, localization, language generators for specific and abstract descriptions.

The loss curves of the experiments are presented in **Figure 5**. Among the proposed cases for plant anomalies description, the model that includes all components (context features, late fusion of the context and region features, and language-localization fusion), as mentioned in *Accurate Localization and Description Section* works better than the others. This can be seen in **Figure 5D**. We may argue that this is mainly because the localization part directly associates the information from both the contextual and region-features and the language generator to estimate the bounding boxes. Each region is associated with a specific sentence. Based on that, we also determined that: a) Context features work for decreasing all losses. b) Late fusion works for decreasing objectness loss relatively. c) Language localization fusion works for stabilizing the bounding box regression loss.

**Figure 6:**
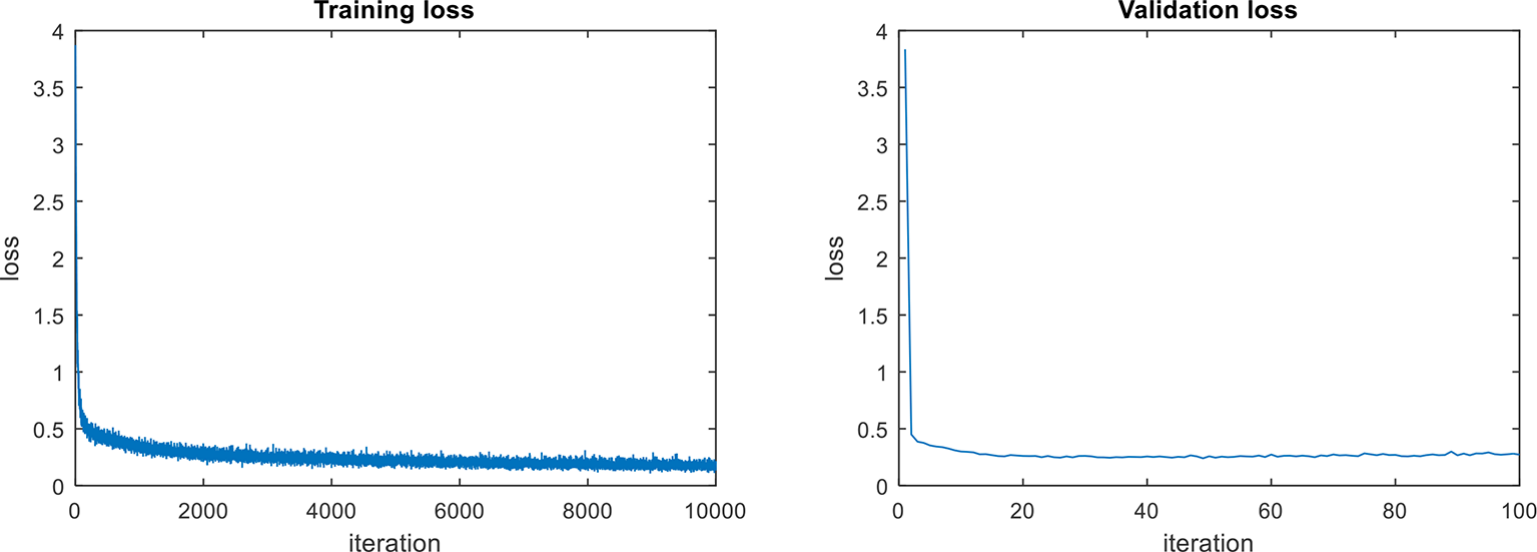
Loss curves of the abstract descriptor (L_abs_). Both curves show a similar trend, which means that there is an adequate performance both during training and validation.

In terms of the object detection loss, our complete model that includes all components, shows an improvement among the models. We assume that the main reason for the outstanding performance of the complete model, compared to other models, is due to the use the context information extracted from the last feature map of the FPN network. The model for abstract description also uses the region features provided by the CNN feature extractor. The training and validation losses of the abstract description are shown in **Figure 6**. Both curves show a similar trend, which means that there is an adequate performance both during training and validation.

**Figure 7:**
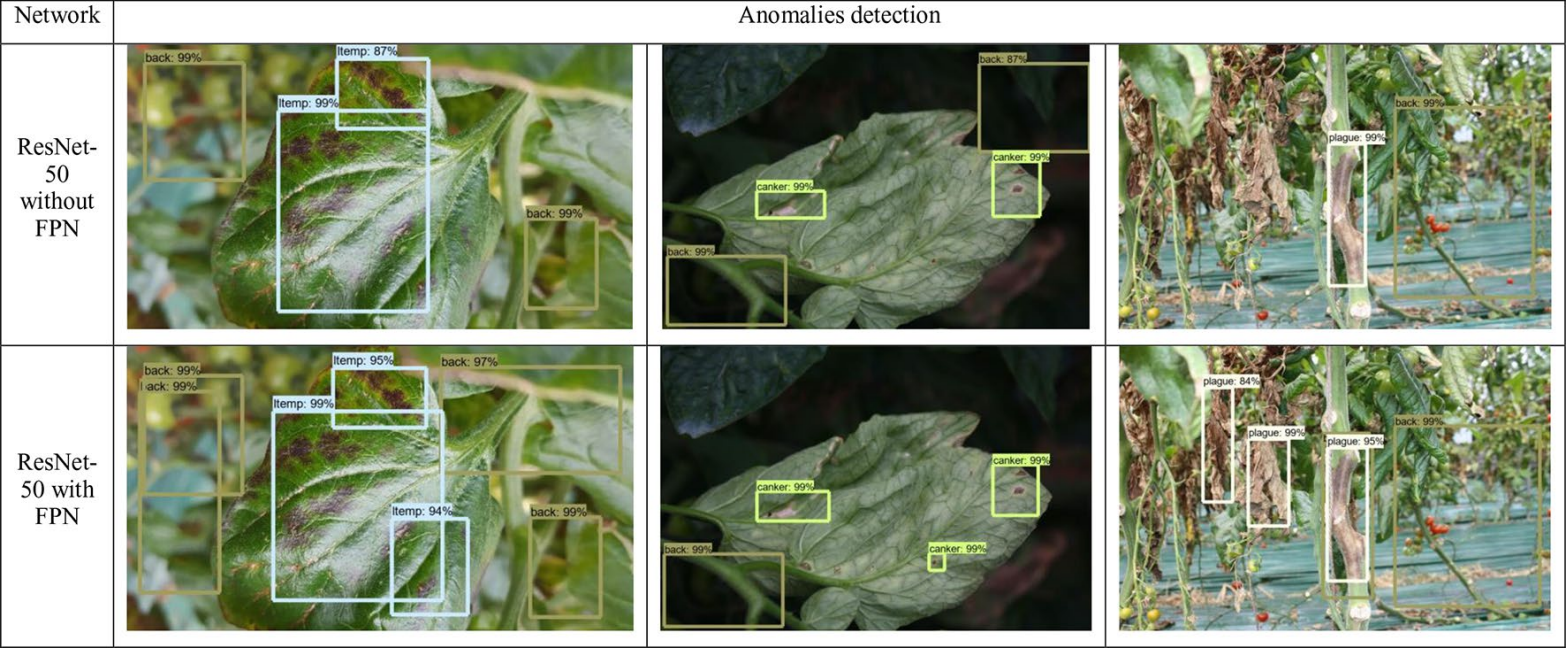
Comparison of some detection results generated by ResNet-50 with/without FPN feature extractors. It shows the capabilities of the FPN-based network to detect objects with small scale and different variations in the images. The names and values in the bounding boxes represent the class and probability of recognition, respectively.

From **Figure 7**, we can also determine the benefits of adding the FPN into the detector. As mentioned above, the challenges of our application demand an object detector that should be able to detect anomalies at various shapes, forms, infection status, and scales, *etc*. In fact, detecting objects at various scales is one of the main difficulties of object detectors. FPN has demonstrated a good response to deal with that specific problem. To demonstrate these capabilities, in **Figure 7**, we show a representation of some anomalies found by the detector. We applied a ResNet-50 with/without FPN and report the detection results. We found that the model which includes a FPN-based architecture is able to correctly find anomalies with small scales, compared to the model without FPN.

**Figure 8:**
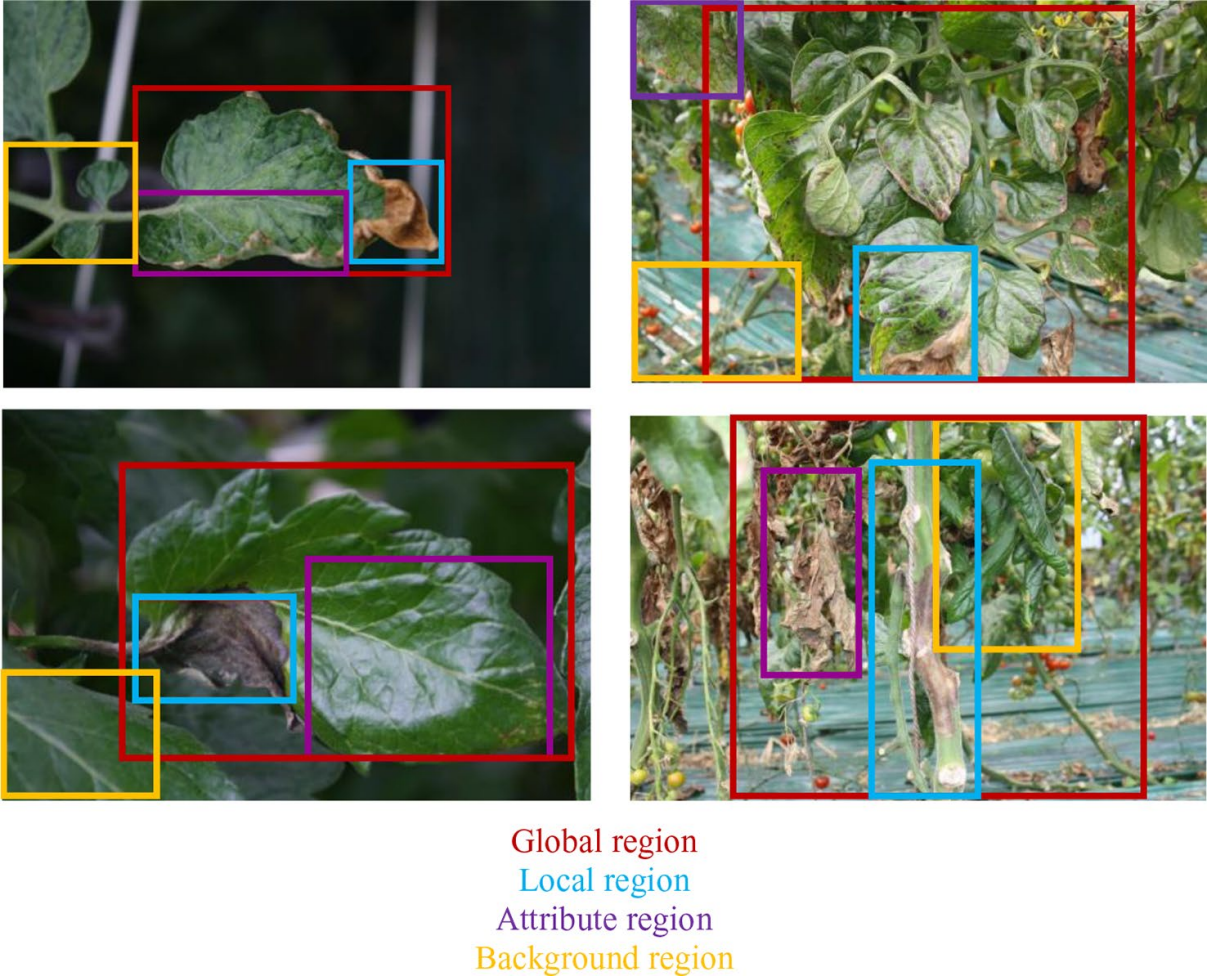
Anomalies are recognized from the region features detected on the given image and their descriptions. We use global and local information to define the anomalies, as well as other attributes, patterns, and context that provide more information about the scene.

**Figure 8** shows some examples of the contextual regions selected by the detector based on FPN. Our detector localizes the regions likely containing anomalies in the plant. We used global and local information to define the anomalies, as well as other attributes, patterns, and context that provide the system with more information about the scene.

**Figure 9:**
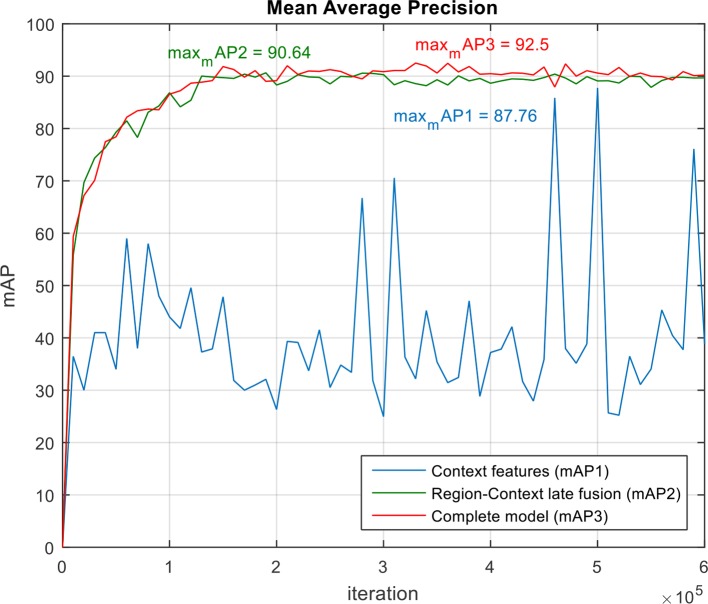
Mean Average Precision (mAP) of our proposed system and its components. Note that our complete model achieves the highest mAP while showing more stability during training.

The performance of the system can be further supported by the mAP results calculated on the validation dataset at different epochs during training. We evaluated our proposed model, including all components previously analyzed, against models that use the components independently. According to the results shown in **Figure 9**, although the model using context features shows a max *mAP o*f 87.76%, it tends to be unstable throughout the iterations. In contrast, using independent LSTM models for the region features and context features respectively, and their subsequent fusion, performs better. Among them, our complete model that includes all components shows more stability while reaching a max *mAP* of 92.5%. Taking all these facts into account, we believe that our proposed model is perfectly adapted to the requirements of our application.

### Qualitative Evaluation

Our experimental results have quantitatively shown the ability of our proposed approach to generate descriptions from the regions containing anomalies in the plant. Here, we qualitatively evaluate how sensitive the system is to generate descriptions using test images.

#### Specific and Abstract Description

**Figure 10:**
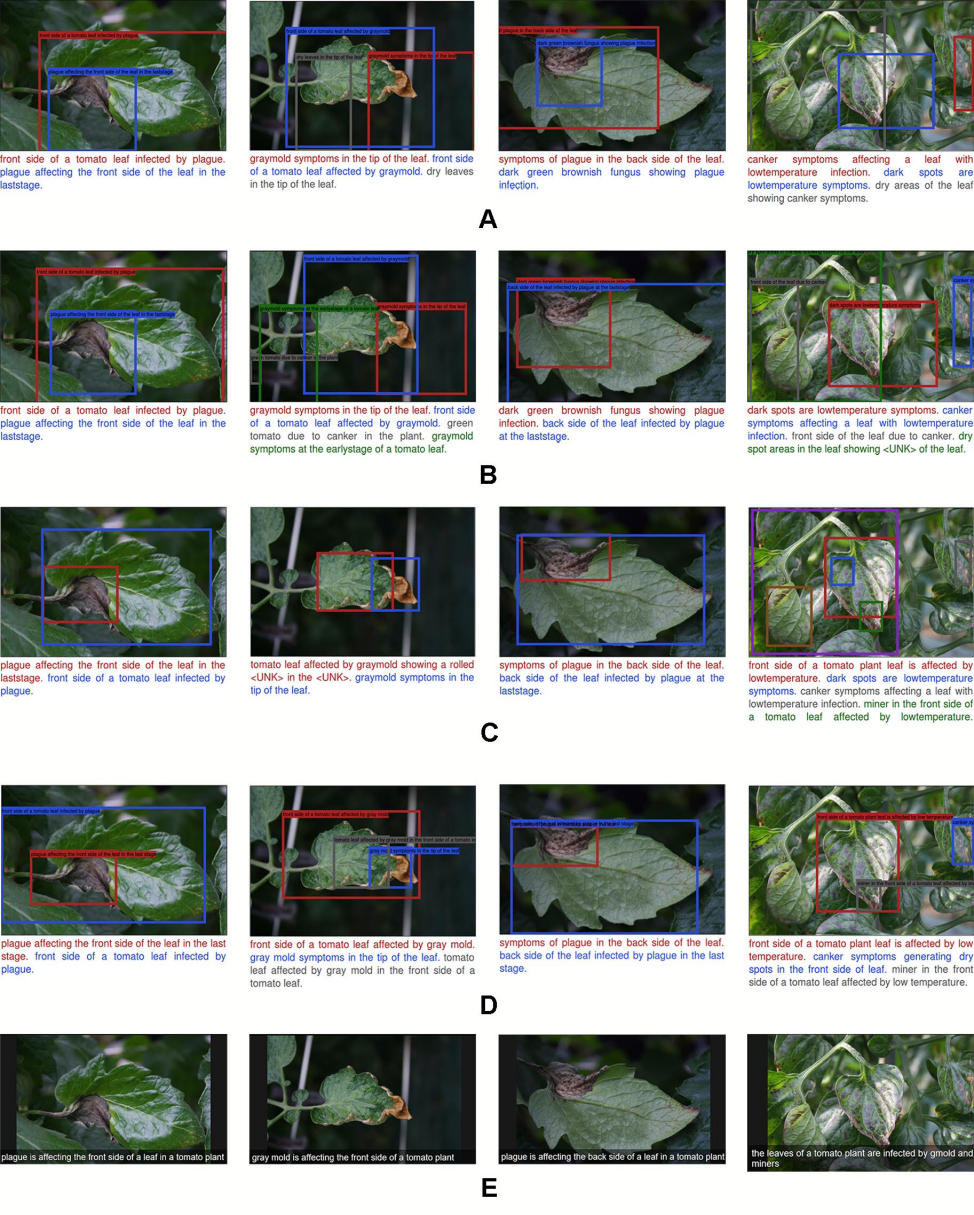
A representation of the qualitative result of the recognition and description of plant anomalies generated by our proposed system. Each row represents the components added to improve the localization and language generation performances. Moreover, we show the specific descriptions **(A**–**D)** of the regions that contain anomalies, and the general abstract description of the scene **(E)**. **(A)** Simple model without context features. **(B)** Model with contextual information. **(C)** Region-context late fusion. **(D)** Complete which includes all components. Our proposed approach generates more detailed and realistic description of the anomalies that affect plants.

Following, we first evaluated the performance of the proposed models using the following objective criteria that focus specifically on the anomalies and their symptoms, as shown in **Figure 10**.

- A model without context features and fusion generates poor performance. It suffers from localization since the bounding boxes cover large areas of the image, including regions of other objects or background. This generates wrong descriptions. See **Figure 10A**.
- The addition of context features from the last layer of the FPN helps to improve performance, especially in terms of objectiveness and language generation. In general, this information considers interactions between anomalies in the image. However, it still lacks precise localization accuracy. See **Figure 10B**.
- The use of independent LSTM modules for the region and context features, respectively, and their subsequent fusion helps to improve the localization of bounding boxes. However, it lacks precision in that it generates wrong sentences and sometimes “Unknown” words that are not included in the vocabulary. We believe that this is because there is no relationship between the bounding boxes location and the words in the sentences. See **Figure 10C**.

We further show in **Figure 10D** some example results of our proposed complete model which includes all the components mentioned above. This model generates a more precise description and localization of the bounding boxes, which in fact may be a consequence of matching the language generator with the location of bounding boxes. Each region of the image is associated with the sentences and keywords. We also show the capabilities of the system to generate a general representation of the scene using an abstract description. To this end, we have taken the information provided by the last convolutional layer of the CNN and use it as the input of the LSTM. With this model, we show that the output information is easier to understand and represents the real state of the plant.

### Ablation Studies

#### Performance of the Loss Function

To understand which functions are critical for the detection and description performances, we analyzed the training results using different combinations of losses. The loss functions are described in *Training Section*. The results are summarized below.

The complete model is trained end-to-end using the loss function mentioned in Equation 6. It includes a specific description loss *L_spec_* abstract description loss *L_abs_* detection loss *L_det_* and bounding box regression loss *L_reg_* To analyze the performance of these losses, we started by looking at the training results using the following combination of losses: 1) Captioning loss, 2) Bounding box regression + Captioning losses, 3) Bounding box regression + Objectness losses, 4) Complete model using all losses (Equation 6).

**Figure 11:**
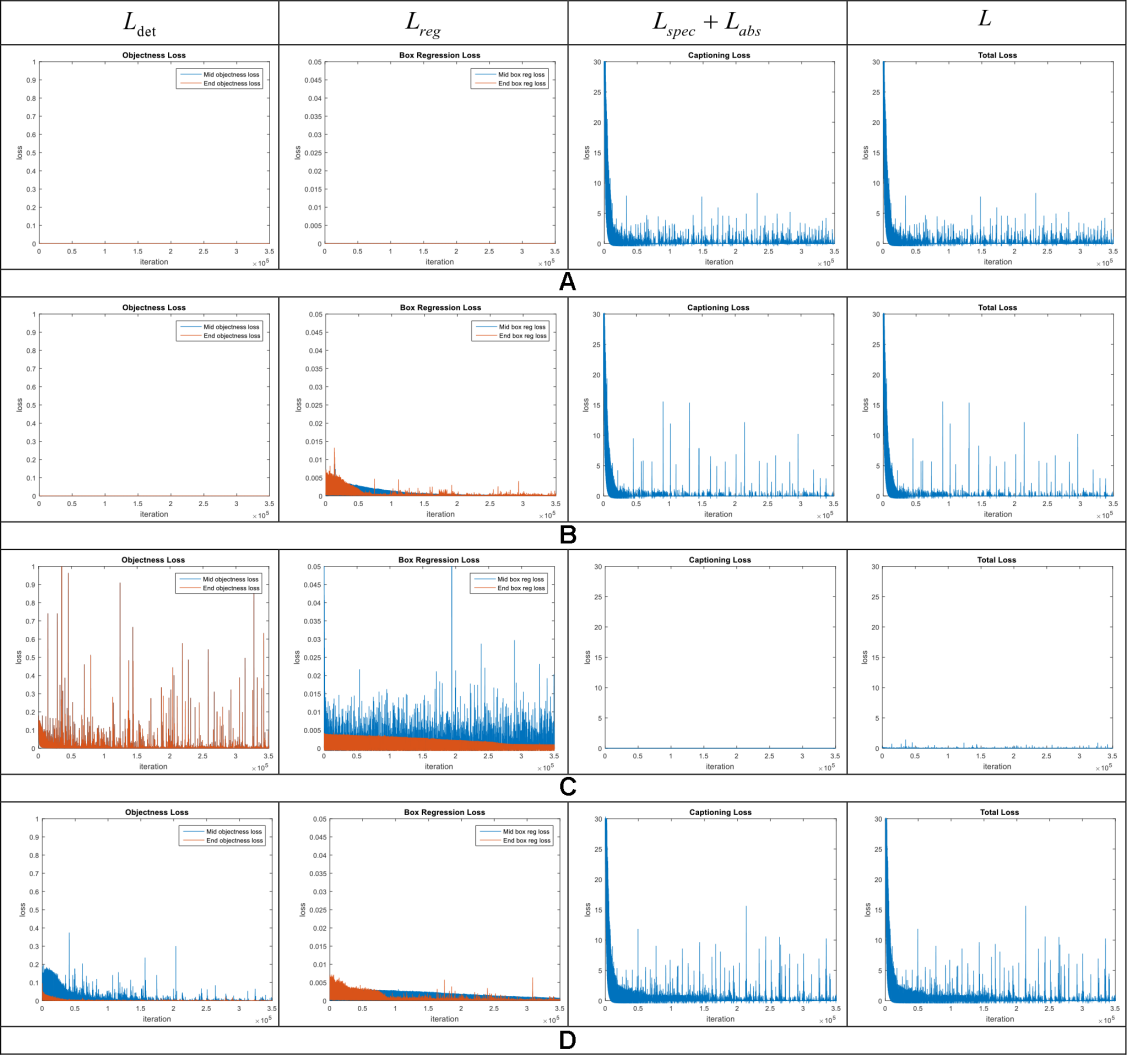
Training curves to evaluate the capabilities of the loss function. Each row represents the curves of different combination losses included in the general framework. **(A)** Captioning loss. **(B)** Bounding box regression loss + captioning loss. **(C)** Bounding box regression loss + objectness loss. **(D)** Model with all losses. Note the system which includes all losses shows better performance while training. Middle means the evaluation at middle layers and End means at the end of the network.

**Figure 11** shows the training curves for the aforementioned cases. Analyzing the results, reveal the importance of both main parts of the model, such as anomalies detection and description. We trained the system using the combinations mentioned above and found that training the system using independent combinations of the losses, generalize worse than combining all losses in the final function. This means that sacrificing some information while training can generally affect the final results in two ways, either in the detector or the language generator. More surprising is that training the model with only the captioning loss (**Figures 11A–B**) can generate an appropriate description but suffers from localization capabilities. On the other hand, training the model with only the bounding box and objectness losses (**Figure 11C**) can generally localize the anomalies but lacks description capabilities such it generates wrong descriptions. In contrast, using a combination of all losses, the system shows a higher score and stability during training. This result is implicitly demonstrating that the efficiency of the language generator widely depends on the efficiency of the detector to localize the anomalies. Much of the representation power comes from the detector which provides an objective localization of the anomalies which are used as input to the language generator. These findings suggest potential benefits of the end-to-end training and the capabilities of each part of the system.

#### Intersection Over Union Threshold on the Detector

**Figure 12:**
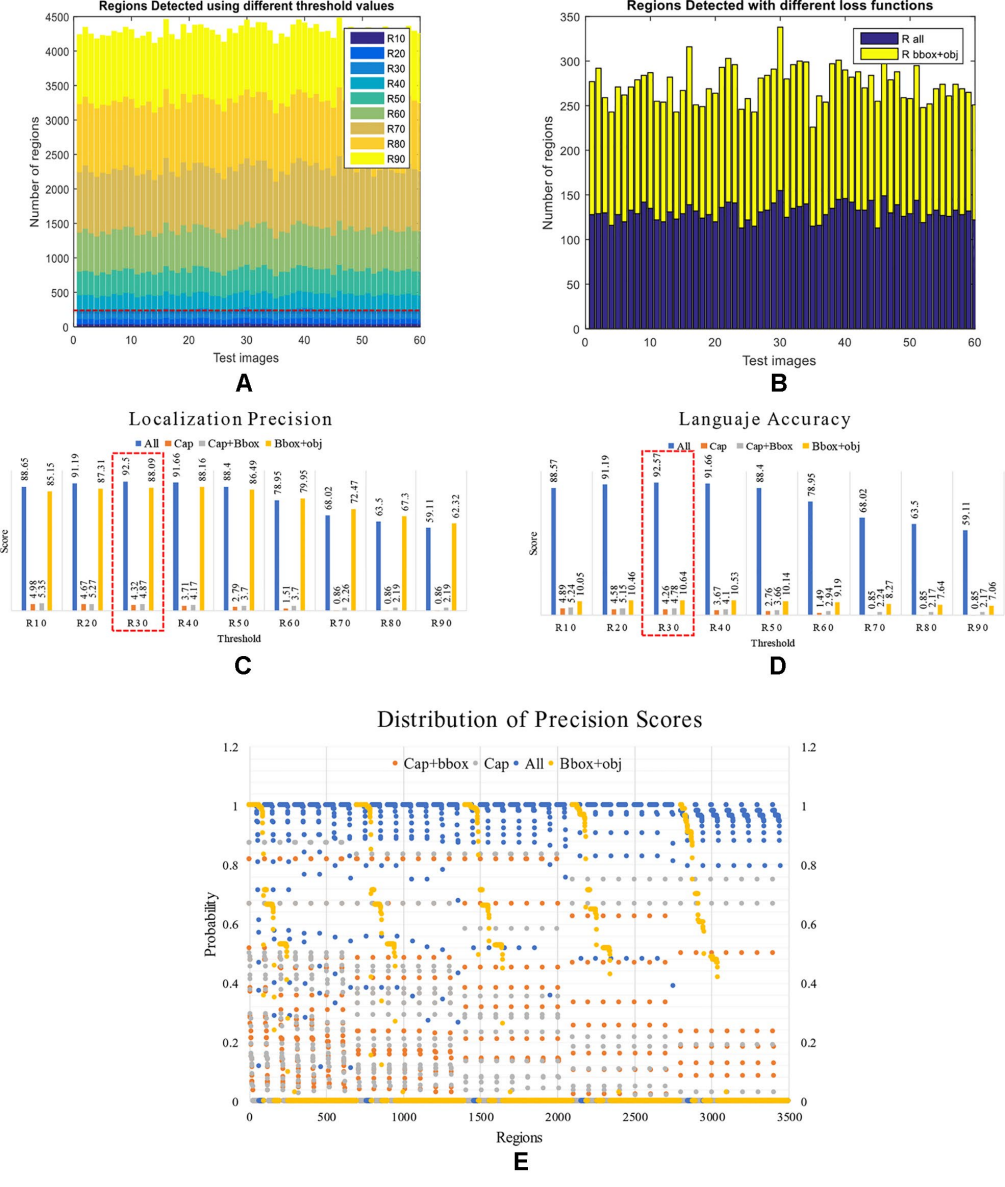
Performance of the detector and language generator. First, we present the number of regions detected by the system under different conditions. **(A)** Using different threshold values. **(B)** With different loss functions. Second, we present a comparison between localization precision and language accuracy. **(C)** Localization precision scores generated by the regions detector. **(D)** Language accuracy scores generated by the LSTM. Each value represents the score generated using different combinations of loss functions at various threshold values. Note that the system performs better when all losses are used such as the case in R30 (3o% IoU threshold). **(E)** Distribution of the precision scores between {0,1} obtained by the detector using different combination of losses. Note that the detector generates a better recognition rate when using all losses, as shown in the probabilities closest to 1.

We further analyzed the results from the detector and its capabilities to identify regions that contain anomalies. We evaluated the performance of the system using different threshold values from 10 to 90% IoU. Previously, an IoU of 50% has been used to evaluate the performance in object detection (Fuentes et al., 2017b). However, in this experiment, our goal was to determine the proper value that fits our approach. We aimed to understand the number of regions necessary for the system to generate accurate results. In average a number of 120 regions per image obtained by using an IoU of 30%, generates the best performance of 92.5% mAP, as shown in **Figure 12A–B**. We determined this value by varying the threshold in the detector and evaluating the number of regions that it generates. Based on this fact, we argue that the performance may be also conditioned to the number of true positives and false positives generated using different IoUs. A representation of the number of regions generated by the detector using different thresholds can be seen in **Figure 12A**.

To support the aforementioned, we further extend this analysis to determine the effect on the number of regions when training the system using a combination of losses as in the previous section. To that effect, we fix the threshold value to 0.3 and train the system to evaluate two specific cases: a) Using the bounding box and objectness losses. b) Using all losses. As shown in **Figure 12B**, in both cases, the detector generates about 120 regions per image on average. This, in fact, demonstrates the capabilities of the detector to localize anomalies that are used as input of the language generator.

#### Localization Precision *vs* Language Accuracy

To support the results of the previous section, we evaluated independently the performance of the localization precision and language accuracy. In **Figures 12C–D**, we show the detailed results of the localization precision (**Figure 12C**), and language accuracy scores (**Figure 12D**). We measured the final results independently in terms of the detection scores and language scores. Although the detector generates satisfactory results, the problem appears when using a combination of some losses. In that case, the mAP can be affected. Using an IoU of 0.3 and all losses to train the system end-to-end, it achieves a detection score of 92.5% and a description score of 92.57%, which in fact, is the best result among the other cases. Certainly, the precision score can be affected using higher threshold values, but in general, using all losses to train the system helps to maintain reasonable performance. **Table 2** shows the mAP scores of the evaluated cases.

**Table 2:** System performance with different combination of losses.

Loss	Detection mAP	Description mAP
All	92.5	92.57
Captioning	4.32	4.26
Bbox reg + Captioning	4.87	4.78
Bbox reg + Objectness	88.09	10.64

To give a sense of the precision, the distribution of the precision scores of the regions generated by the system is also presented in **Figure 12E**. This representation shows the probability of regions between {0,1}. We showed that a combination of all losses achieved a higher number of regions with higher probability, which is significantly ahead of the other cases.

## Conclusion

In this paper, we introduced an efficient end-to-end diagnostic system that automatically recognizes plant anomalies along with their location in the image, and allows to generate more detailed information about their symptoms and interactions with the scene. It uses an image as input and generates a diagnostic result shown as a set of bounding boxes and glocal descriptions as output. Our system consists of two main parts, first, a detector is trained to obtain a set of region features that contain anomalies of the plants using a Region-based Deep Neural Network based on a FPN architecture, and then a language generator takes the features of the detector as input and generates descriptive sentences of the symptoms using Long-Short Term Memory (LSTM). Our loss metric allows the system to be trained end-to-end from the object detector to the language generator. We also demonstrated that the use of context information and fusion techniques provide a substantial improvement in the localization and description parts while making the training process more stable. For the purpose of this work, we created a new dataset called Tomato Plant Anomalies Description Dataset, that specifically includes three types of information: bounding box coordinates of the anomalies, detailed description of the anomalies within the regions and, abstract description of the scene. In general, our experimental results showed that, compared to previous diseases detection systems, our work provides more objective information of the anomalies. It does not only locate the anomalies in the images but also describes in detail the symptoms of those anomalies using sentences that are easier to be interpreted by the users. In addition, an objective evaluation may also allow users to understand the relationships between pathologies and their evolution throughout their stage of infection, location in the plant, symptoms, *etc*. The model estimates visual and semantic correspondences for all the evaluated cases, even for complex objects such as miners, leaf mold located in the front and backside of the leaves, plague affecting different parts of the plant, and cases with more than one disease affecting the leaves. The generated sentences are clear and grammatically correct while providing concise information about the pathogens that affect the tomato plants. We presented a cost-efficient tool that provides farmers with a technology that facilitates proper handling of crops. Furthermore, we hope that this approach will serve as a reference guide to facilitate future research in the area of precision agriculture, as well as in the design of more efficient monitoring systems to control plant anomalies, as the application could be extended to other crops.

## Data Availability Statement

The datasets for this manuscript are not publicly available because this research has been carried out as part of a project funded by the Government of Korea. We have a contract not to open the dataset until the project is concluded. Any question regarding the data set should be directed to Alvaro Fuentes, afuentes@jbnu.ac.kr. .

## Author Contributions

AF designed the system, performed the experiments, and wrote the paper. DP and SY advised in the design of the system and analyzed the strategies to find the best method for efficient plant anomalies description.

## Funding

This work was supported in part by Basic Science Research Program through the National Research Foundation of Korea(NRF) funded by the Ministry of Education (No. 2019R1A6A1A09031717), and with the support of "Cooperative Research Program for Agriculture Science and Technology Development (Project No. PJ01389105)" Rural Development Administration, Republic of Korea.

## Conflict of Interest

The authors declare that the research was conducted in the absence of any commercial or financial relationships that could be construed as a potential conflict of interest.
